# Study of the Scale Effect on the Mechanical Properties of High-Strength Concrete

**DOI:** 10.3390/ma18163795

**Published:** 2025-08-13

**Authors:** Marek Miazgowicz, Lucyna Domagała

**Affiliations:** 1 CUT Doctoral School, Faculty of Civil Engineering, Cracow University of Technology, 31-155 Kraków, Poland; 2 Faculty of Civil Engineering, Cracow University of Technology, 31-155 Kraków, Poland; lucyna.domagala@pk.edu.pl

**Keywords:** modulus of elasticity, compressive strength, high-strength concretes, dynamic modulus of elasticity, scale effect

## Abstract

This paper presents the effect of specimens’ shape and size on the modulus of elasticity and compressive strength of high strength concrete. The European Standard EN 12390-13 allows not only for different procedures but also for different shapes and sizes of test specimens. However, it does not provide a relationship between specimen size and shape and elastic modulus. The aim of the research was to determine the influence of the shape and size of specimens on the measured values of the secant and dynamic modulus of elasticity and compressive strength. The analysis was carried out on cube and cylindrical specimens of various sizes and slenderness. Concrete with the mean strength of f_cm,cyl_ = 101.9 MPa was used for the tests. The research used 64 specimens of various sizes and shapes. Compared to the results obtained for the basic cylindrical specimen (150 × 300), the differences reached 19% for E_d_ and 14% for E_c,s_. The results indicate that in the case of the tested composite the key factor influencing the value of the elastic modulus and compressive strength of specimens is its individual structure that determines its density, while the scale and shape of the specimens have less effect on the mechanical properties.

## 1. Introduction

Since 2013, a new European Standard EN 12390-13 [1] has been in force concerning the testing of the modulus of elasticity of concrete. It provides specimens of various sizes and shapes. In addition to basic cylindrical specimens (with a diameter of 150 mm and a height of 300 mm), which according to the previous guidelines, were commonly used as the only ones, the current standard also provides for testing the modulus of elasticity on other cylindrical and cube specimens. The standard EN 12390-13 [1] states that the size and shape of the specimen may affect the value of the modulus of elasticity but does not specify how. Based on the literature review, it was found that there are few available studies and no guidelines regarding the effect of scale and shape of specimens in the study of the modulus of elasticity of structural concrete.

The aim of this research was to verify the effect of size and shape of concrete specimens on the secant and dynamic modulus of elasticity and compressive strength. This article presents the factors influencing the value of the modulus of elasticity of concrete, the causes of scale effects, and the available research on this topic. The research, results, and conclusions are then presented.

Determining the modulus of elasticity on specimens other than basic cylindrical specimens is widely used in practice, e.g., in situations where specimens are taken directly from the structure for diagnostic purposes, in the case of prefabricated elements of smaller dimensions, or when using concrete on aggregate with dimensions exceeding 43 mm. For example, when testing the modulus of elasticity of structural concrete for water dams, larger cube specimens with a base width of 45 cm and a height of 90 cm are used because the maximum aggregate size can be as large as 120 mm.

The main components of concrete are aggregate, cement, and water, as well as possible admixtures, additives, or fibres. The mechanical properties of concrete depend on the type of aggregate and cement and the value of the water–cement ratio. These factors significantly affect the modulus of elasticity. The use of a higher cement class increases the compressive strength of concrete, which results in an increase in the modulus of elasticity. According to [2], the same relationship occurs for concrete classes, the higher the Young’s modulus.

The type of aggregate used, its grain size, and quantity are undoubtedly some of the most important factors affecting the modulus of elasticity. The European Standard EN-1992-1-1 [3] describes the dependence of the type of aggregate on Young’s modulus, but ignores grain size, which is also important. The research conducted in [4] shows that the effect of grain size is more noticeable in the case of concretes with a fraction of 0/8 mm than 0/16 mm. It was found that concrete with a grain size of 0/8 mm and basalt aggregate can achieve a modulus of elasticity about 20% higher than concrete with limestone aggregate. In the case of concretes with a fraction of 0/16 mm, this difference was less than 5%. The European Standard EN-1992-1-1 [3] gives average values of elastic moduli for different classes of concrete, but they apply to concrete with quartz aggregate. It also describes the influence of the type of aggregate, such as limestone, sandstone, or basalt, on the modulus of elasticity. Unfortunately, there are no guidelines for concretes made of granite aggregate. Modification of the modulus of elasticity for concretes based on aggregates other than quartzite is as follows:

- A 10% reduction in value in the case of limestone aggregate,
- A 30% reduction in value in the case of sandstone aggregate,
- A 20% increase in value in the case of basalt aggregate.

However, as noted in [5,6], when considering the influence of aggregate on the modulus of elasticity, it should not be limited only to the type. As shown in [7], the influence of aggregate grain size is more visible for concretes containing fractions of 0/8 mm than 0/16 mm.

The modulus of elasticity is also significantly affected by the value of the water–cement (w/c) ratio. As shown by studies [8], a lower value of Young’s modulus is obtained for concretes with a higher w/c ratio. In the case of reducing the w/c ratio from 0.70 to 0.58, an increase in the modulus of elasticity by 9% was obtained; however, the same change also caused an increase in compressive strength by as much as 31%. In addition, the modulus value can also be influenced by the specimen moisture content. As shown in [9,10], in the case of normal-weight concretes, the modulus of elasticity is higher for wet specimens than for dry ones. As we can see in [11,12], this influence is even greater in the case of lightweight concretes due to their higher porosity. Additionally, as shown in the studies in [13], the specimen moisture also affects the sound pulse velocity, which is the basis for determining the dynamic modulus of elasticity.

The standard EN 12390-13 [1] allows for two methods of testing the modulus of elasticity: A and B. However, it does not specify the relationship between them. In [14], an analysis of the influence of both methods on the value of the modulus of elasticity of concrete was presented. It was found that, regardless of the type of aggregate and cement paste in concrete, Method A yields higher results than Method B. It is possible that the difference is due to the strengthening and compression of the concrete specimen caused by the initial loads applied in Method A.

The scale effect in testing concrete specimens refers to the phenomenon in which the mechanical properties of concrete, such as compressive strength, modulus of elasticity, or other parameters, may change depending on the size of the specimens being tested. This is an important phenomenon in materials engineering, because concrete is characterized by a non-homogeneous structure, consisting of different components (cement, aggregate, water, additives), which may have different effects on its mechanical properties depending on the scale. In practice, this means that the properties observed on smaller concrete specimens may not be directly applicable to larger structural elements. The smaller the specimen, the more decisive is the aggregate size and adhesion, which affects the homogeneity of the concrete.

According to theories from the field of fracture mechanics and probability theory, we can obtain different values of the constant characteristic quantities of the material, depending on the specimen size. Gryfith’s theory explains that defects (e.g., pores) occur in every material. As shown in [15,16] under the influence of load, defects lead to the occurrence of high stress concentrations in the material, i.e., very high stresses are reached in small areas of the specimen, which initiates the destruction process, while the average stress in the entire element is relatively low.

Griffith’s hypothesis is applied to tensile failure, but it can be extended to cases of compression failure, because even when the principal stresses are compressive, the micro-crack stresses are tensile at certain points, so that rupture can occur. Similarly, Weibull’s statistical strength theory [17] is based on the assumption that defects occur in every material. Their distribution in the material is identical. The weakest point determines the failure [18]. Therefore, the larger the specimen, the greater the probability of a critical defect in it, which will initiate the failure process. This is illustrated by the Weibull Formula (1), which determines the probability of failure depending on the specimen volume. The Weibull modulus m is related to the probability of a critical defect. The smaller its value, the greater the spread of the material strength values.

(1) $$p_{f}=1-exp\left[-V_{0}{\left(\frac{\sigma_{i}-\sigma_{u}}{\sigma_{0}}\right)}^{m}\right]$$

$p_{f}$—probability of brittle decohesion of a specimen of volume *V* subjected to uniform tensile stress *σ*,
$V_{0}$—specimen volume,
$\sigma_{0}$—characteristic quantity for which the probability of survival is 1/e,
$\sigma_{u}$—threshold value below which specimens cannot be destroyed,
*m*—Weibull modulus.

Therefore, the scale effect is more noticeable in the case of material with greater inhomogeneity.

There are many publications in the scientific literature describing the scale effect in strength tests, e.g., [19,20]. According to the studies conducted in [4], both compressive and tensile strengths obtain higher values for smaller specimens. It was shown there that for very large specimens, the size effect starts to decrease. This is due to the homogeneity of concrete, which is greater when testing large specimens, as well as the probability of the occurrence of the largest critical defect (the weakest link). From a certain specimen size, it statistically occurs in almost every specimen. This effect is less visible for high-strength concretes. This is due to the greater porosity of lower-strength concretes.

According to [21], the scale effect in strength tests is more noticeable in the case of normal strength concretes than in the case of lightweight concretes.

The only article found addressing this issue [22] was analyzed. The authors compared the obtained values of moduli of elasticity on cylindrical specimens of two sizes: 150 mm in diameter and 300 mm in height and 100 mm in diameter and 200 mm in height. The tests were carried out on concretes with target compressive strengths of 30, 35, and 40 MPa and after 4, 7, 14, and 28 days. The obtained results do not clearly determine the effect of specimen scale on the modulus of elasticity. On average, larger specimens obtained a 1.4% higher secant modulus of elasticity, and 1.3% higher ultrasonic wave velocity values were recorded for smaller specimens, which resulted in their higher dynamic modulus of elasticity. However, these differences are smaller than the calculated coefficients of variation. The smallest effect of specimen size on the modulus of elasticity was noted in the concrete of the lowest strength and in tests after 4 and 7 days. However, there are no studies on the effect of specimen shape on the modulus of elasticity.

Therefore, tests were carried out to check the relationship between the value of the elastic modulus and the shape and size of the specimen.

## 2. Materials

**Figure 1:**
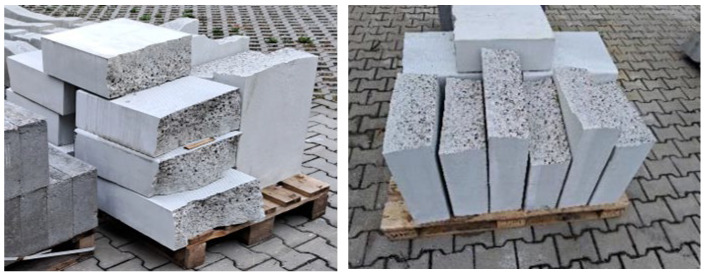
Precast concrete elements from which the specimens were made. The prefabricated elements were supplied by a private manufacturer who does not provide a detailed design of the concrete mix.

Prefabricated concrete elements were selected for the tests, shown in Figure 1. They were made of high-strength concrete. They were seasoned for about one year, so that the test time had no effect on specific features. The concretes were made on 2/8 mm gravel aggregate and 8/12 mm crushed limestone. The specimens were cut out one year after the elements had been formed so that the duration of testing such a large population of specimens did not affect the results of determining the mechanical characteristics.

**Figure 2:**
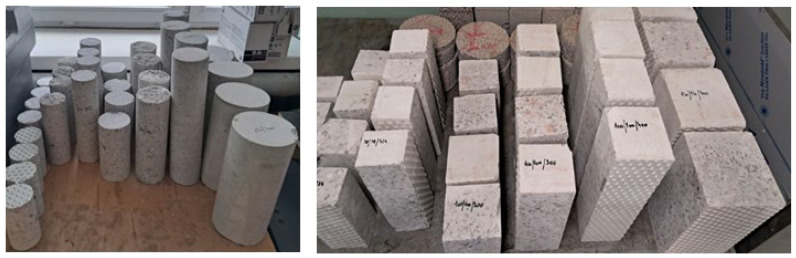
Cylindrical and cube specimens for testing. The 80 × 160 mm specimens were drilled both perpendicularly and parallel to the forming direction. The remaining specimens were drilled perpendicularly to the forming direction of the precast elements.

From the prepared precast elements, a set of 64 specimens was drilled—32 cylindrical and 32 cube—shown in Figure 2.

As shown in Table 1 and Table 2, the tests were carried out on 8 series of cylindrical specimens and 8 series of cube specimens. Each series consisted of 4 specimens, where one was used to determine the initial strength, and the others were used to test the modulus of elasticity. In order to verify the direction of concrete forming, two series of cylindrical specimens with a diameter of 80 mm and a height of 160 mm and two series of cube with a width of 80 mm and a height of 160 mm were prepared, drilled perpendicularly and parallel to the direction of forming.

The tests were carried out on specimens in the air-dry state, because this is the most common form of testing specimens taken from structures. According to the research conducted in [23], the influence of moisture content when testing high-strength concrete can be considered negligible.

## 3. Methods

Before starting the elastic modulus tests, the densities of the specimens were determined in accordance with the standard EN 12390-7 [24]. Companion specimens were prepared from the same batch of concrete and used to determine the compressive strength. After testing the elastic modulus, compressive strength tests were carried out in accordance with the standard EN 12390-3 [25]. The tests were carried out in the laboratory of the Cracow University of Technology on a testing machine manufactured by ZwickRoell and compliant with the standard EN 12390-4 [26], according to which the compressive strength was also determined.

**Figure 3:**
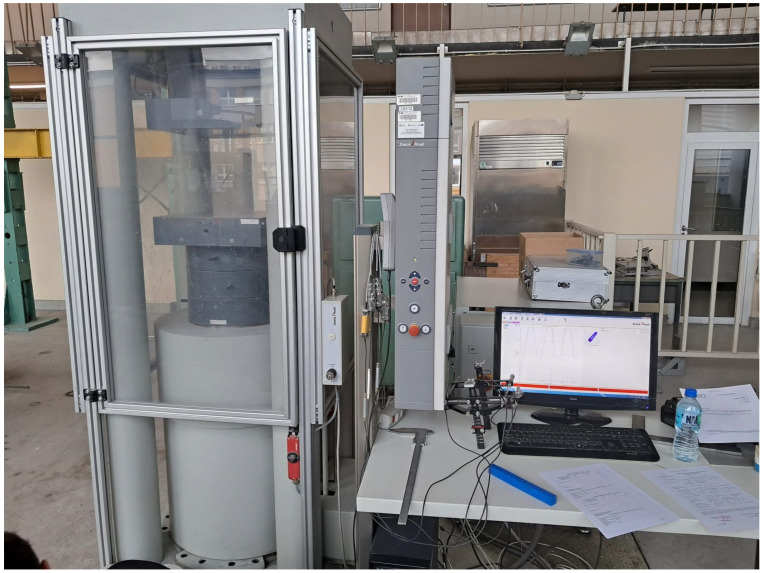
Test of secant modulus of elasticity.

**Figure 4:**
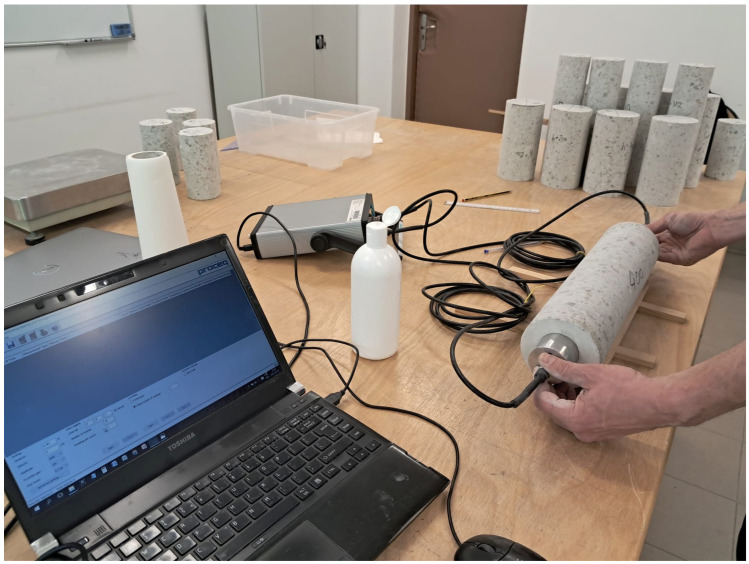
Tests of dynamic modulus of elasticity.

As shown in Figure 3 and Figure 4, tests of the secant and dynamic modulus of elasticity and compressive strength were carried out.

For the purpose of determining the dynamic modulus of elasticity, the ultrasonic method was used. According to the standard EN 12504-4 [27], this method consists of measuring the velocity of an ultrasonic wave of a given frequency in a material of a given density. Ultrasonic testing equipment consists of an electric pulse generator, a pair of probes, an amplifier, and an electronic synchronizing device for measuring the time interval between the beginning of the pulse generated on the transmitting probe and the beginning of its arrival at the receiving probe. The measurement consists of placing the probes (transmitting and receiving) on opposite bases of the concrete specimen and measuring the path of the wave and the time it takes for it to pass through the material. The longer the time, the greater the discontinuities in the material structure, which results in a lower value of the modulus of elasticity. For specimens up to 50 mm long, high-frequency probes (60 kHz to 200 kHz) are used. In the conducted tests, the pulse length was 6.1 μs, and the probe frequency was 82 kHz. The dynamic modulus of elasticity Ed is calculated from Formula (2), in accordance with the ASTM C 215 [28] standard.

(2) $$E_{d}=\frac{{V_{P}}^{2}\cdot \rho \cdot \left(1+\nu \right)\cdot \left(1-2\cdot \nu \right)}{\left(1-\nu \right)}$$

$E_{d}$—dynamic modulus of elasticity;
$V_{P}$—wave velocity;
ρ—concrete density;
*ν*—Poisson’s ratio.

The secant modulus of elasticity tests were conducted using Method B, according to the standard EN 12390-13 [1]. It allows for analysis on cylindrical and cube specimens whose diameter or width is at least 3.5 times greater than the coarsest aggregate fraction, and the length to width or diameter ratio meets the condition 2 ≤ L/d ≤ 4. This standard allows for the use of specimens formed and taken from the structure but does not specify the number of specimens required for testing. It is suggested that the specimens be stored at a temperature of about 20 degrees Celsius, with a tolerance of ±2 °C. 

**Figure 5:**
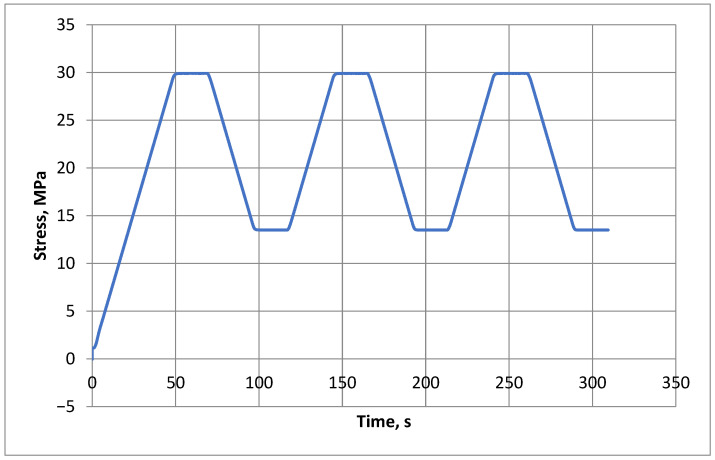
Graph showing the loading cycles for the selected test specimen.

As seen in Figure 5, the specimen is subjected to three main loading cycles, during which the stress is gradually increased. The stress level should be maintained for a period of no longer than 20 s. After the third cycle, the stabilized secant modulus of elasticity is determined according to Formula (3).

(3) $$E_{c,s}=\frac{\sigma_{a}^{m}-\sigma_{b}^{m}}{\varepsilon_{a,3}-\varepsilon_{b,2}}$$

$E_{c,s}$—stabilized secant modulus of elasticity;
$\varepsilon_{a,3}$ i $\varepsilon_{b,2}$—strains measured at the third cycle corresponding to the measured stress values $\sigma_{a}^{m}$ i $\sigma_{b}^{m}$.

After the measurements have been carried out, it is necessary to gradually increase the load on the specimen until it is destroyed in order to determine the compressive strength of the concrete.

## 4. Results

### 4.1. Dynamic Tests

**Figure 6:**
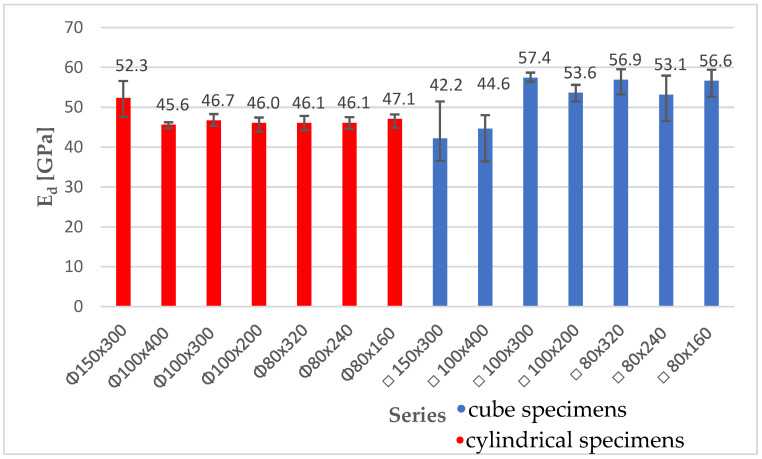
Summary of average values of the dynamic elastic modulus for cube and cylindrical specimens.

First, the dynamic modulus of elasticity was tested using ultrasonic pulse velocity measurement, in accordance with the standard EN 12504-4 [27]. Analysis of the obtained results showed an average of 3% higher ultrasonic pulse velocity values for cube specimens compared to cylindrical ones. For most specimens, a tendency of increasing E_d_ values was observed for specimens with the same cross-sectional area and lower height. However, in several cases, the scale effect was not visible. This may be related to the large variation in specimen density and high standard deviations and coefficients of variation. Figure 6 presents a summary of the dynamic modulus of elasticity values for the tested series. No significant scale effect was observed, but the influence of the specimen shape was visible. Cube specimens obtained an average of 10% higher dynamic modulus of elasticity value, compared to cylindrical specimens. E_d_ values for cylindrical specimens were in the range of 45.6–52.3 GPa, while for cube specimens, they were in the range of 42.2–57.4 GPa. The coefficients of variation in the tested specimen series were in the range of 1–9%. They were calculated based on Formula (4).

(4) $$V=\frac{s}{\bar{x}}\cdot 100\%$$

*V*—coefficients of variation;
*s*—standard deviation;
$\bar{x}$—arithmetic mean of the values obtained on specimens in a given series.

Standard deviations calculated according to Formula (5).

(5) $$s=\sqrt{\frac{\sum {\left(x-\bar{x}\right)}^{2}}{n}}$$

*s*—standard deviation;
x—subsequent data values obtained for individual specimen of the series;
$\bar{x}$—arithmetic mean of the values obtained on specimens in a given series;
n—number of specimens in a given series.

The graph shows error bars illustrating the maximum and minimum values obtained for a specimen in a given series.

Table 3 compares the average dynamic values (E_d_) of each series with the average value obtained for the basic cylindrical specimen (150 × 300 mm) E_d,cyl_.

**Figure 7:**
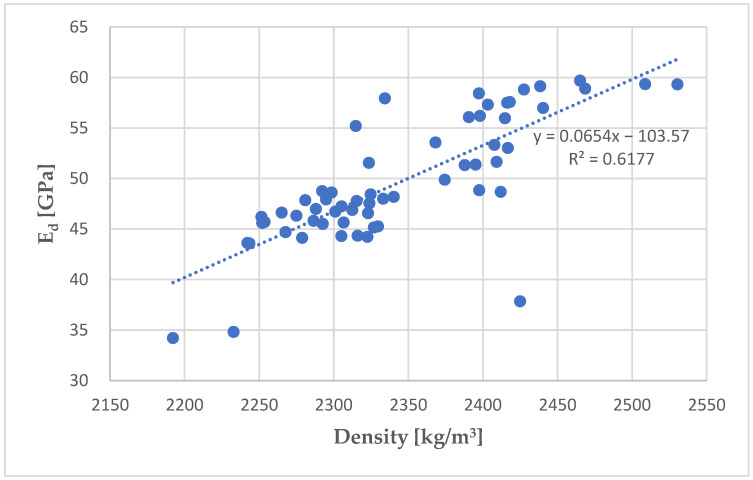
Dependence of the dynamic modulus of elasticity on the density of concrete.

The effect of concrete density on the dynamic modulus of elasticity was also analyzed. For most specimens, a trend towards higher ultrasonic pulse velocities and, therefore, higher E_d_ values was observed, as shown in Figure 7. This tendency is related to the lower porosity of specimens with higher density. Pores as defects refract the path of the passing ultrasonic pulse, which prolongs its passage time through the specimen.

### 4.2. Static Tests

**Figure 8:**
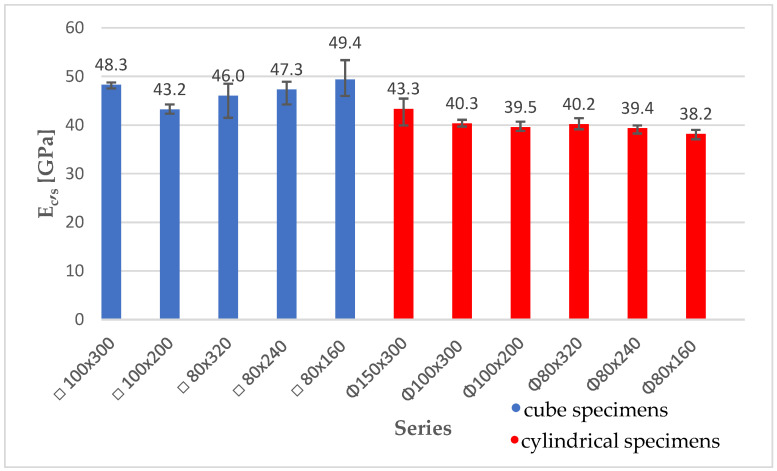
Summary of average values of the secant stabilized elastic modulus for cube and cylindrical specimens.

The secant stabilized modulus of elasticity tests were conducted using Method B, according to the standard EN 12390-13 [1]. These tests allowed for the verification of the grinding technology, which proved ineffective with high specimens (400 mm) using the equipment available. Therefore, tests on 400 mm high specimens were omitted at this stage. Also, due to exceeding the permissible dimensional deviations of the 150 × 300 mm cube specimens, the secant modulus of elasticity tests were not conducted on them. The analysis of the obtained test results indicates that on average 16% higher results were obtained on cube specimens (E_c,s_ in the range of 43.2 GPa–49.4 GPa) in relation to cylindrical specimens (E_c,s_ in the range of 38.4 GPa–43.3 GPa). However, due to the large variation in the density of concrete of individual specimens, this result must be subjected to further verification. As a result of this differentiation, the cube specimens had a 4% higher average density than the cylindrical specimens, which may be the key reason for the higher E_c,s_ values. The coefficients of variation in the individual series in the secant stabilized modulus of elasticity test were in the range of 1–7% and, therefore, comparable to the case of the dynamic modulus of elasticity tests. As shown in Figure 8, the scale effect was observed only for cube specimens with a width of 80 mm and heights of 160, 240, and 320 mm, where the E_c,s_ value was in the range of 46.0 GPa–49.4 GPa. With the increase in the height of these specimens, the modulus of elasticity decreased by 4% and 7%, respectively. It should be noted that the specimens of this series showed similar density. This suggests that the relationship between modulus of elasticity and specimen size is similar to the relationship presented in [4] between strength and specimen size. The graph shows error bars illustrating the maximum and minimum values obtained for a specimen in a given series.

Table 4 compares the values of the secant stabilized modulus of elasticity of the individual series with the value obtained for the basic cylindrical specimen (150 × 300 mm) E_c,s,cyl_.

**Figure 9:**
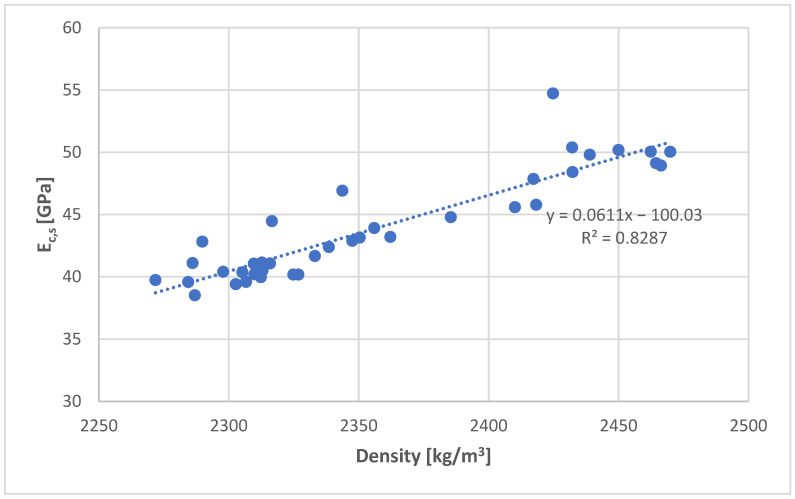
Dependence of the secant modulus of elasticity on the density of concrete.

Similar to the dynamic modulus tests, the density of the tested specimens proved to be crucial. Noticeably higher modulus values (E_c,s_) were recorded for higher density concretes, as shown in Figure 9. With the increase in density by 100 kg/m^3^, the E_c,s_ value increased by an average of 6.1 GPa. The coefficient of variation in the density test for all specimens was 3%.

### 4.3. Compressive Strength Tests

In the last stage of the tests, the compressive strength of the specimens was determined. Strength tests carried out on basic cylindrical specimens (150 × 300 mm) indicate an average compressive strength of 101.9 MPa; therefore, the tested concrete should be classified as high-strength concrete.

**Figure 10:**
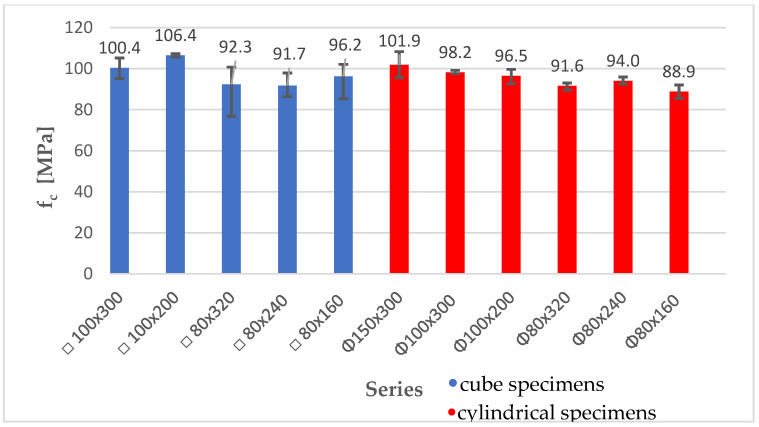
Summary of average compressive strength values for cube and cylindrical specimens.

As shown in Figure 10, the average compressive strength of all series was in the range of 88.9 MPa–106.4 MPa. The coefficients of variation in the individual series reached up to 12%. Such a dispersion of the strength results is mainly caused by differences in the density of the specimens, resulting from the forming of the prefabricated elements used for the tests. The graph shows error bars illustrating the maximum and minimum values obtained for a specimen in a given series.

Table 5 compares the strength of individual series with the strength of the basic cylindrical specimens f_cm,cyl_.

## 5. Discussion

- For the high-strength concrete tested, no significant scale effect was observed either in the elastic modulus or compressive strength tests.
- The concrete used for the tests was characterized by particularly high strength. In its analysis, cracks appear only at a very high level of stress, while during tests, in accordance with the standard EN 12390-13:2021 [1], the specimen is subjected to maximum stresses equal to only 1/3 of the compressive strength. Therefore, when testing the secant modulus of elasticity, the scale effect for high-strength concretes may be less noticeable than in the case of ordinary concretes.
- The conducted studies have shown that the key factor influencing the value of the modulus of elasticity and the compressive strength of the specimens is concrete structure, which determines the density. In connection with this, due to the differences in the density of individual specimens, it is also difficult to identify the scale effect in the case of the compressive strength test.
- It is worth noting that the coefficient of variation in the density test was significantly lower than in the tests of the dynamic and secant modulus of elasticity. It turned out to be the highest in the compressive strength test.
- The authors intend to conduct further research in which they will standardize the specimens in terms of density by making prefabricated concrete elements themselves.

## Figures and Tables

**Table 1:** Cylindrical specimen series.

Series Number	Diameter (d) [mm]	Height (h) [mm]
Φ80 × 160	80	160
Φ80 × 160’	80	160
Φ80 × 240	80	240
Φ80 × 320	80	320
Φ100 × 200	100	200
Φ100 × 300	100	300
Φ100 × 400	100	400
Φ150 × 300	150	300

**Table 2:** Cube specimen series.

Series Number	Width (a, b) [mm]	Height (h) [mm]
□80 × 160	80	160
□80 × 160’	80	160
□80 × 240	80	240
□80 × 320	80	320
□100 × 200	100	200
□100 × 300	100	300
□100 × 400	100	400
□150 × 300	150	300

**Table 3:** Comparison of the dynamic modulus of elasticity of individual series.

Series	Density [kg/m^3^]	E_d_ [Mpa]	E_d_/E_d,cyl_
Φ80 × 160	2310	47.1	90.1%
Φ80 × 240	2300	46.1	88.1%
Φ80 × 320	2320	46.1	88.1%
Φ100 × 200	2260	46.0	88.0%
Φ100 × 300	2280	46.7	89.3%
Φ100 × 400	2270	45.6	87.2%
Φ150 × 300	2370	52.3	100.0%
□80 × 160	2450	56.6	108.2%
□80 × 240	2390	53.1	101.5%
□80 × 320	2400	56.9	108.8%
□100 × 200	2400	53.6	102.5%
□100 × 300	2410	57.4	109.8%
□100 × 400	2330	44.6	85.3%
□150 × 300	2350	42.2	80.7%

**Table 4:** Comparison of the secant stabilized modulus of elasticity of individual series.

Series	Density [kg/m^3^]	E_c,s_ [Mpa]	E_c,s_/E_c,s,cyl_
Φ80 × 160	2310	38.2	88.2%
Φ80 × 240	2300	39.4	91.0%
Φ80 × 320	2320	40.2	92.8%
Φ100 × 200	2260	39.5	91.2%
Φ100 × 300	2280	40.3	93.1%
Φ150 × 300	2370	43.3	100.0%
□80 × 160	2450	49.4	114.1%
□80 × 240	2390	47.3	109.2%
□80 × 320	2400	46.0	106.2%
□100 × 200	2400	43.2	99.8%
□100 × 300	2410	48.3	111.5%

**Table 5:** Comparison of the strength of individual series.

Series	Density [kg/m^3^]	f_cm_	f_cm_/f_cm,cyl_
Φ80 × 160	2310	88.9	87.2%
Φ80 × 240	2300	94.0	92.2%
Φ80 × 320	2320	91.6	89.9%
Φ100 × 200	2260	96.5	94.7%
Φ100 × 300	2280	98.2	96.4%
Φ150 × 300	2370	101.9	100.0%
□80 × 160	2450	96.2	94.4%
□80 × 240	2390	91.7	90.0%
□80 × 320	2400	92.3	90.6%
□100 × 200	2400	106.4	104.4%
□100 × 300	2410	100.4	98.5%

## Data Availability

Data are contained within the article.
